# Supplementary figures and images for: Functionalizing silica sol–gel with entrapped plant virus-based immunosorbent nanoparticles
Source: J Nanobiotechnology. 2022 Mar 4;20:105. doi: 10.1186/s12951-022-01303-1 (PMC8895542; doi:10.1186/s12951-022-01303-1)

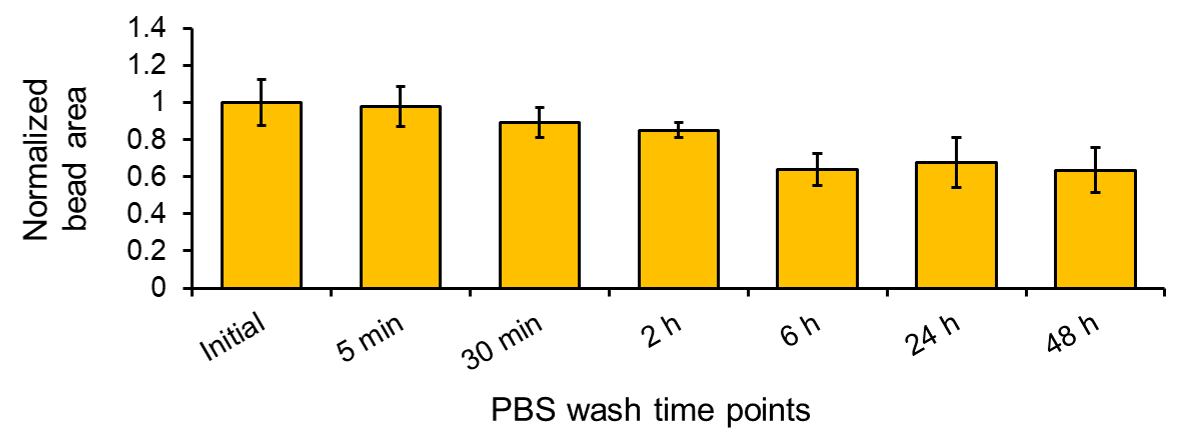

Supplement: Supplementary file 1 — Additional file 1: Figure S1. Bead area over time (Cy5-TMV, 2 µL bead). Normalized bead area over time for 2 µL volume silica bead containing Cy5-TMV over 48 h. Beads were exchanged into fresh PBS buffer after each measurement. Error bars represent one standard deviation with biological triplicate. [file 12951_2022_1303_MOESM1_ESM.png]

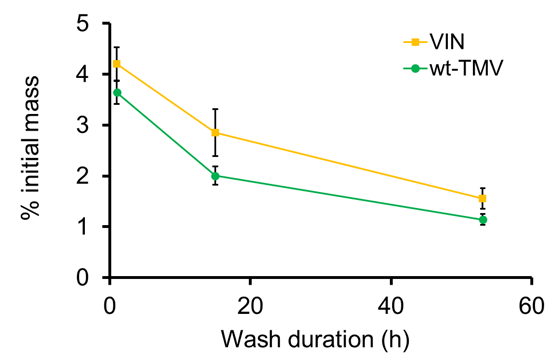

Supplement: Supplementary file 2 — Additional file 2: Figure S2. Absorbance readings during equilibration (VIN and wt-TMV, 40 µL bead). UV–vis A280 measurements of the PBS wash solution over ~ 2 days of equilibration reported as a fraction of the initial A280 measurement for the VIN or wt-TMV added into the silica sol–gel synthesis. The PBS wash solution was collected at each sample timepoint and exchanged with fresh buffer. This experiment was conducted using 1 volume PBS wash solution per volume silica sol–gel to improve limit of detection. The retention of the PBS wash solution in the buffer exchange (~ 50% total wash solution) dictates that these results represent an upper bound of the initial mass lost during washing. Error bars represent one standard deviation using biological triplicate [file 12951_2022_1303_MOESM2_ESM.png]
